# Association of leptin and insulin-like growth factor-1 gene polymorphisms with milk production and reproductive traits in Indonesian Friesian Holstein cattle

**DOI:** 10.14202/vetworld.2026.2264-2278

**Published:** 2026-06-05

**Authors:** Ahmad Pramono, Muhammad Cahyadi, Aprilya Hanifah Tamartian, Hesti Fatonah, Imelda Renita Alfara, Sahrul Romadhon, Farouq Heidar Barido

**Affiliations:** 1 Department of Animal Science, Faculty of Animal Science, Universitas Sebelas Maret, Surakarta 57126, Indonesia; 2 Halal Research Center and Services (HRCS), Institute for Research and Community Service, Universitas Sebelas Maret, Surakarta 57126, Indonesia

**Keywords:** dairy cattle genetics, fertility traits, Friesian Holstein cattle, insulin-like growth factor-1, leptin gene, marker-assisted selection, milk production, single nucleotide polymorphism

## Abstract

**Background and Aim:**

Genetic polymorphisms in candidate genes associated with metabolic regulation and reproductive physiology are increasingly used to support marker-assisted selection in dairy cattle. Among these, leptin (*LEP*) and insulin-like growth factor-1 (*IGF-1*) genes have been linked to milk production and fertility traits in several bovine populations. However, information regarding the association of these polymorphisms with productive and reproductive performance in Indonesian Friesian Holstein cattle remains limited. Therefore, this study investigated the association of *LEP* (g.820 C>T and g.1127 A>T) and *IGF-1* (c.309 G>T and c.335 A>T) polymorphisms with milk production and reproductive traits in Indonesian Friesian Holstein cattle.

**Materials and Methods:**

A total of 710 Friesian Holstein cows maintained at a commercial dairy farm in West Java, Indonesia, were used in this study. Genomic DNA was isolated from whole blood samples and genotyped using polymerase chain reaction-restriction fragment length polymorphism assays. Genotype and allele frequencies, polymorphic information content, and Hardy-Weinberg equilibrium were determined for each single nucleotide polymorphism. Associations between genotypes and productive traits, including days in milk, 305-day milk yield, total yield, average yield, and peak yield, as well as reproductive traits including calving interval, days open, first insemination postpartum, service per conception, and conception rate, were analyzed using mixed-effects models.

**Results:**

The *LEP* g.820 C>T polymorphism exhibited moderate polymorphism and significant associations with days in milk, calving interval, first insemination postpartum, days open, and conception rate (p < 0.05). Cows carrying the CC genotype showed superior reproductive performance and higher conception rates compared with TT genotype carriers. Conversely, *LEP* g.1127 A>T showed limited polymorphism and no significant association with productive or reproductive traits (p > 0.05). The *IGF-1* c.309 G>T locus was monomorphic, whereas c.335 A>T was polymorphic but showed no significant associations with milk production or reproductive parameters (p > 0.05).

**Conclusion:**

The *LEP* g.820 C>T polymorphism demonstrated significant associations with economically important productive and reproductive traits and may serve as a valuable molecular marker for marker-assisted selection in Indonesian Friesian Holstein cattle. In contrast, the evaluated *IGF-1* polymorphisms showed limited utility for genetic selection in this population.

## INTRODUCTION

Indonesia’s dairy industry is predominantly based on Friesian Holstein (FH) cattle because of their superior genetic potential for milk production, with average lactation yields capable of reaching 15–20 L/day during peak production in the second month postpartum [1]. Despite this genetic potential, domestic fresh milk production in Indonesia reached only 968,980 tons in 2022 [2], fulfilling merely 20% of the national demand of approximately 4.4 million tons and resulting in nearly 80% dependence on imported dairy products [3]. In addition, milk consumption remains relatively low at 16.23 kg/capita/year, largely due to limited domestic supply and economic constraints. This substantial gap between genetic potential and actual productivity indicates that current production systems have not fully optimized the genetic capacity of Indonesian FH cattle, although heritability contributes approximately 30% to milk yield variation under environmental influences [4].

Current dairy cattle breeding programs in Indonesia follow the Indonesian National Standard (SNI 2735:2014), which mainly focuses on phenotypic evaluation of morphometric and production-related traits while giving limited attention to genomic-based selection strategies [4, 5]. Conventional selection approaches for economically important polygenic traits, such as milk production and reproductive efficiency, generally require long generation intervals and delayed phenotypic observations, thereby slowing genetic progress. Marker-assisted selection (MAS) has emerged as a promising alternative approach because it integrates DNA polymorphisms with phenotypic records to improve selection accuracy at an earlier age [6], reduce generation intervals [7], and accelerate genetic improvement in dairy populations [8]. Previous studies have identified several candidate genes associated with milk production and reproductive performance, including *LEP* and *IGF-1* [9–11]. However, the majority of these studies were conducted in temperate dairy populations, whereas limited information is available regarding their associations in tropical-adapted Indonesian FH cattle raised under commercial production systems. Moreover, genotype × environment (G×E) interactions may influence allelic effects under Indonesia’s specific climatic, nutritional, and management conditions, potentially causing differences in gene-trait associations compared with those observed in temperate regions.

Although polymorphisms of the *LEP* and *IGF-1* genes have been extensively investigated in several dairy cattle populations worldwide, evidence regarding their associations with milk production and reproductive traits in Indonesian FH cattle remains scarce. Most previous studies were performed under temperate environmental conditions and involved genetically distinct dairy populations, which may not accurately represent tropical dairy production systems. In addition, environmental stressors, nutritional management, climatic adaptation, and breeding practices in Indonesia may influence the expression and phenotypic effects of these polymorphisms through G×E interactions. Consequently, the applicability of previously reported genetic markers in Indonesian dairy herds remains uncertain. Furthermore, no previous study has comprehensively evaluated the combined associations of *LEP* g.820 C>T, *LEP* g.1127 A>T, *IGF-1* c.309 G>T, and *IGF-1* c.335 A>T polymorphisms with both productive and reproductive traits in Indonesian FH cattle maintained under commercial dairy farm conditions. This lack of population-specific molecular information limits the implementation of genomic-assisted breeding strategies for improving dairy cattle productivity and reproductive efficiency in Indonesia.

The *IGF-1* gene encodes insulin-like growth factor-1, a peptide hormone involved in cellular proliferation, differentiation, metabolic regulation, ovarian follicular development, oocyte maturation, embryogenesis, and fertility maintenance [5–8]. Polymorphisms within regulatory regions, particularly in the 5′-untranslated region (5′-UTR), have been associated with differences in milk production and reproductive performance among dairy cattle breeds [9]. Similarly, the *LEP* gene encodes leptin, an adipocyte-derived hormone that regulates appetite, energy balance, metabolic homeostasis, and reproductive signaling through hypothalamic pathways [11]. Variations within promoter, intronic, and coding regions of *LEP* may alter gene expression and physiological responses, thereby affecting milk yield, calving interval, conception rate, feed efficiency, and growth traits in dairy cattle [12–15].

Therefore, this study aimed to evaluate the associations between *LEP* polymorphisms (g.820 C>T/Sau3AI and g.1127 A>T/ClaI) and *IGF-1* polymorphisms (c.309 G>T/AluI and c.335 A>T/ApoI) with milk production and reproductive traits in Indonesian FH cattle. Specifically, this study investigated genotype and allele frequencies, Hardy-Weinberg equilibrium status, and the effects of these polymorphisms on productive traits, including milk yield parameters and reproductive performance indicators under commercial dairy management conditions in Indonesia. The findings of this study are expected to provide scientifically validated molecular genetic information that may support the development of genomic-assisted and precision breeding strategies for sustainable genetic improvement of dairy cattle productivity and reproductive efficiency in Indonesia.

## MATERIALS AND METHODS

### Ethical approval

All experimental protocols and animal care procedures involving 710 FH cows were rigorously reviewed and approved by the Ethical Clearance Commission of the Faculty of Veterinary Medicine, Universitas Gadjah Mada, Indonesia (Approval Number: 057/KE/FKH-Eks/2022; approved on March 15, 2022). The study was conducted in accordance with Indonesian national animal research regulations [Permentan No. 42/Permentan/OT.140/7/2020] and internationally accepted animal welfare guidelines. Blood collection procedures were performed using BD Vacutainer® K2-EDTA tubes (Becton Dickinson, Franklin Lakes, NJ, USA) during routine herd health management activities. Individual animal restraint was maintained for <2 min by trained veterinarians and technical personnel to minimize handling stress. Numerical pain scores averaged 1.2 ± 0.4/5 and were characterized only by mild ear flicking and tail movement responses. Before sampling, all cows underwent veterinary clinical examination, and animals showing mastitis, lameness, metabolic disorders, or abnormal body condition score (BCS) values were excluded from the study. Only clinically healthy cows with BCS ranging from 2.5 to 3.5/5 were included for molecular and phenotypic analyses.

### Study period and location

This study was conducted from July to November 2024 at PT Ultra Peternakan Bandung Selatan (UPBS), a commercial dairy farm located in Bandung Regency, West Java, Indonesia. The farm operates under ISO 22000:2018 certification standards for dairy production and herd management. Laboratory analyses were carried out at the Integrated Laboratory Unit, Biology Sub-Laboratory, Universitas Sebelas Maret, Surakarta, Indonesia.

### Study design

A cross-sectional molecular association study was performed using blood samples collected from 710 FH cows maintained under commercial dairy production systems. Animals were identified using radio frequency identification (RFID) ear tags following the protocol described by Setiawanti *et al*. [16]. The study population consisted of two independent genotyping cohorts, including 330 samples for *LEP* single nucleotide polymorphisms (SNPs) (g.820 C>T and g.1127 A>T) and 380 samples for *IGF-1* SNPs (c.309 G>T and c.335 A>T). The non-overlapping subsampling approach was implemented because of DNA yield optimization requirements for multilocus and single-locus polymerase chain reaction-restriction fragment length polymorphism (PCR-RFLP) analyses.

All cows exhibited standard FH phenotypic characteristics, including a white forehead blaze and black-and-white piebald coat coloration. Animals were maintained under confined free-stall housing systems with approximately 30 m²/cow lying space and received Total Mixed Ration (TMR) diets consisting of a 65:35 forage-to-concentrate ratio, with daily dry matter intake ranging from 18 to 20 kg/cow/day. Feed was provided once daily at 07:00, and water was available *ad libitum* through automatic drinking systems. Artificial insemination breeding programs were conducted using semen from 42 elite sires, of which approximately 68% possessed the TT genotype for the *IGF-1* c.335 A>T polymorphism according to farm breeding records.

### Sample collection protocols and DNA extraction

Blood samples were aseptically collected from the coccygeal vein (*Vena caudalis*) at the tail base of 710 FH cows using 21G × 1” BD Vacutainer® K2-EDTA tubes (Becton Dickinson, Franklin Lakes, NJ, USA) at a sampling volume of 5 mL/animal (<0.5% body weight). Sampling was performed by certified veterinarians during routine herd examinations between 07:00 and 09:00 to minimize physiological stress, with individual restraint duration maintained at <2 min. Numerical pain scores averaged 1.2 ± 0.4/5 and were limited to mild ear flicking responses.

Samples were transported at 4°C within <4 h to the on-site laboratory facility at UPBS and immediately processed for genomic deoxyribonucleic acid (DNA) extraction using the Wizard® Genomic DNA Purification Kit (Promega, Madison, WI, USA) according to the manufacturer’s protocol optimized for bovine whole blood. Briefly, 300 μL blood was mixed with 900 μL Cell Lysis Solution and homogenized for 10 min at 26^o^C, followed by centrifugation at 10,000 × *g* for 20 s. The supernatant was discarded, and the white blood cell pellet was resuspended in 300 μL Nuclei Lysis Solution by gentle inversion. Subsequently, 100 μL Protein Precipitation Solution was added, vortexed for 20 s, and centrifuged at 10,000 × *g* for 3 min. The nucleic acid-containing supernatant was transferred to a clean tube, followed by isopropanol precipitation using 300 μL isopropanol and gentle inversion. After centrifugation for 1 min, the DNA pellet was washed using 300 μL of 70% ethanol and centrifuged again for 1 min. DNA pellets were air-dried for 15 min at RT and rehydrated in 100 μL DNA Rehydration Solution overnight at 4°C. The extraction procedure yielded genomic DNA concentrations of 85.3 ± 22.1 ng/μL with A260/A280 ratios of 1.82 ± 0.04, indicating DNA quality suitable for PCR-RFLP analyses.

### PCR-RFLP genotyping

PCR-RFLP genotyping targeted four SNP loci, including *LEP* g.820 C>T/Sau3AI, *LEP* g.1127 A>T/ClaI, *IGF-1* c.309 G>T/AluI, and *IGF-1* c.335 A>T/ApoI, using National Center for Biotechnology Information (NCBI)-validated primers (Table 1). Amplification reactions were performed using a Bio-Rad T100™ Thermal Cycler (Bio-Rad Laboratories, Hercules, CA, USA) in a final reaction volume of 25 μL containing 12.5 μL GoTaq® Green Master Mix (Promega, Madison, WI, USA), 1 μL forward primer (10 μM), 1 μL reverse primer (10 μM), 1 μL genomic DNA template (85.3 ± 22.1 ng/μL), and 9.5 μL nuclease-free H_2_O. The GoTaq® Green Master Mix contained 2 mM MgCl_2_, 0.2 mM deoxynucleotide triphosphates (dNTPs), and 1 U Taq DNA polymerase.

**Table 1:** Designed primers used to amplify *LEP* and *IGF-1* genes.

Gene	Polymorphism	Primer sequence (5′–3′)	Annealing temperature (°C/s)	Product size (bp)	Restriction enzyme	Reference
*LEP*	g.820 C>T	F: TGGAGTGGCTTGTTATTTTCTTCT	36/30	422	Sau3AI	[1]
		R: GTCCCCGCTTCTGGCTACCTAACT				
*LEP*	g.1127 A>T	F: GATTCCGCCGCACCTCTC	37/30	467	ClaI	[19]
		R: CCTGTGCAAGGCTGCACAGCC				
*IGF-1*	c.309 G>T	F: TCCCACTCTAAAGCTAGGCC	58/30	290	AluI	
		R: GCTCAGCCTCATAACTCCGA				
*IGF-1*	c.335 A>T	F: CCCTGGAGTTGGTAGATTGC		234	ApoI	
		R: CCCTGGAGTTGGTAGATTGC				

Thermal cycling conditions consisted of an initial denaturation step at 95°C for 3 min, followed by 35 amplification cycles of denaturation at 95°C for 30 s, SNP-specific annealing temperatures ranging from 55.2°C to 59.8°C according to primer requirements (Table 1), extension at 72°C for 45 s, and a final extension at 72°C for 7 min. Amplified products were verified by electrophoresis using 2% agarose gels, producing expected amplicon sizes of 422 bp for g.820 C>T, 467 bp for g.1127 A>T, 290 bp for c.309 G>T, and 234 bp for c.335 A>T (Figure 2 and Figure 3).

Restriction digestion reactions were prepared in 15 μL reaction mixtures containing 5 U FastDigest® restriction enzymes (New England Biolabs, Ipswich, MA, USA), including Sau3AI, ClaI, AluI, and ApoI, together with CutSmart® Buffer. Digestion was performed at 37°C for 2 h, except for Sau3AI digestion, which was conducted overnight. Enzymes were heat-inactivated at 80°C for 20 min. Digested fragments were separated using 2% agarose gel electrophoresis in 0.5× Tris-borate-ethylenediaminetetraacetic acid (TBE) buffer at 90 V for 45 min and visualized using GelRed® staining under a Bio-Rad ChemiDoc MP imaging system (Bio-Rad Laboratories). Genotypes were assigned according to characteristic fragment digestion patterns.

### Statistical analysis of associations

Data analysis involved calculation of genotype and allele frequencies followed by Hardy-Weinberg equilibrium (HWE) testing using the Chi-square (χ²) goodness-of-fit test. The HWE model was used to evaluate whether observed genotype frequencies deviated significantly from expected frequencies under assumptions of random mating, absence of selection, mutation, migration, and genetic drift. The equilibrium condition was mathematically expressed as follows [17]:

p + q = 1

p² + 2pq + q² = 1

where pp and qq represent allele frequencies within the population, p² and q² represent expected frequencies of homozygous genotypes, and 2pq represents the expected frequency of heterozygous genotypes. The Chi-square test compared observed genotype distributions with expected HWE proportions to determine whether evolutionary or population effects influenced each locus.

χ² = (O − E)² / E

The Chi-square (χ²) value was calculated using the formula above, where O represents observed genotype frequencies and E represents expected genotype frequencies under HWE conditions.

Associations between genotypes and reproductive and milk production traits were analyzed using MINITAB software version 19.1 (Minitab LLC, State College, PA, USA). In the statistical model, genotype was considered a fixed effect, whereas sire was treated as a random effect to account for genetic relatedness among animals. Reproductive and productive traits were included as dependent variables, while lactation number was incorporated as a covariate to minimize potential confounding effects. Multiple comparisons among genotype means were subsequently evaluated using Tukey’s Honestly Significant Difference (HSD) test. Statistical significance was declared at p < 0.05.

## RESULTS

### DNA extraction

**Figure 1:**
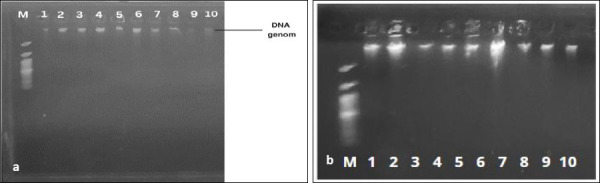
Visualization of DNA extraction results: (a) *Leptin* gene and (b) *IGF-1* gene. M = Marker ladder 100 bp, (1–10) = Sample.

High-molecular-weight genomic DNA was successfully extracted from a total of 710 FH cow blood samples, as verified by 1% agarose gel electrophoresis (Figure 1). The *LEP* cohort exhibited intact genomic smears with estimated fragment sizes ranging from 10 to 20 kb, showing 100% integrity without detectable degradation. Meanwhile, the *IGF-1* cohort demonstrated consistent high-quality profiles with DNA concentrations of 85.3 ± 22.1 ng/μL (range: 42-142 ng/μL), A260/A280 ratios of 1.82 ± 0.04 (1.74-1.92), and A260/A230 ratios of 2.1 ± 0.2 (1.8-2.4). PCR suitability criteria were fulfilled by 98.2% of samples, whereas 12 faint bands still exceeded the minimum amplification threshold. These publication-grade quality parameters confirmed DNA integrity suitable for reliable downstream PCR-RFLP genotyping of *LEP* (g.820 C>T and g.1127 A>T) and *IGF-1* (c.309 G>T and c.335 A>T) polymorphisms [18, 19].

### PCR amplification

**Figure 2:**
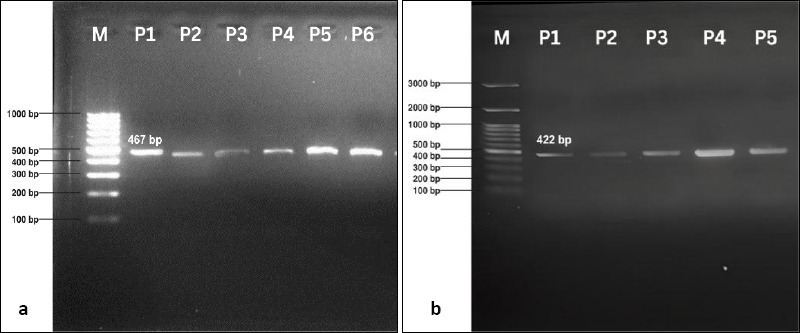
Visualization of the PCR products of the Leptin gene: (a) SNP g.820 C>T; (b) SNP g.1127 A>T. M = Marker ladder 100 bp, (P1–P6) = Sample.

PCR amplification of the *LEP* gene targeting the SNP g.820 C>T consistently generated an expected amplicon size of 422 bp, as confirmed by electrophoretic visualization (Figure 2a). Similarly, amplification of the SNP g.1127 A>T produced a fragment of 467 bp corresponding precisely to the designed primer annealing sites and expected product length (Figure 2b). Optimization of annealing temperature was identified as a critical parameter for achieving specific and efficient amplification because it directly influenced primer-template hybridization fidelity and subsequent target DNA amplification efficiency.

**Figure 3:**
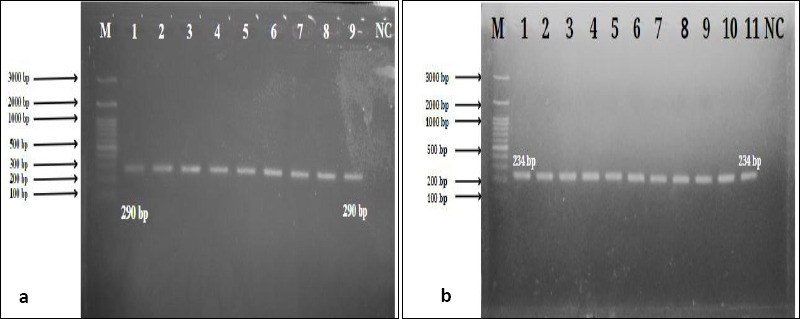
Visualization of PCR products for the IGF-1 gene: (a) SNP c.309 G>T, (b) SNP c.335 A>T. M = Marker ladder. NC = Negative control.

Regarding the *IGF-1* gene, primer specificity and amplification efficiency were validated by the consistent presence of discrete electrophoretic bands corresponding to theoretical fragment sizes. PCR amplification generated fragments of 290 bp for SNP c.309 G>T (Figure 3a) and 234 bp for SNP c.335 A>T (Figure 3b). Both amplicons were below the 300 bp threshold and consistent with previous reports describing *IGF-1* gene fragments of approximately 249 bp, thereby confirming the robustness and reproducibility of the PCR protocol used in this study. These fragment sizes facilitated high-resolution genotyping and demonstrated the precision of primer design and thermal cycling conditions for polymorphism detection associated with genetic association analyses [20].

### PCR-RFLP analysis

**Figure 4:**
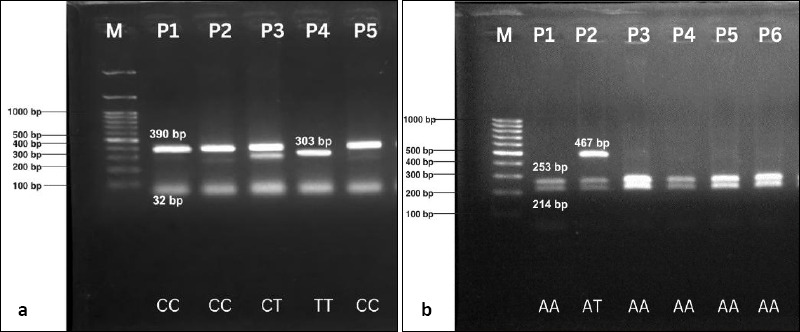
Visualization of PCR-RFLP products for the Leptin gene: (a) SNP g.820 C>T, (b) SNP g.1127 A>T. M = Marker ladder, (P1–P6): Sample.

PCR-RFLP analysis of the *LEP* gene at the g.820 C>T locus (Sau3AI digestion, n = 330) revealed three distinct genotypic classes corresponding to C/T allelic variants, as visualized by 2% agarose gel electrophoresis (Figure 4a). The homozygous CC genotype (75.2%, n = 248) generated characteristic fragments of 390 bp and 32 bp because of complete Sau3AI digestion at two restriction sites. The heterozygous CT genotype (23.3%, n = 77) exhibited a composite fragment pattern comprising 390 bp, 303 bp, 88 bp, and 32 bp, indicating partial digestion associated with heterozygous restriction sites. The homozygous TT genotype (1.5%, n = 5) yielded 303 bp, 88 bp, and 32 bp fragments, confirming the absence of the C allele restriction site.

The *LEP* g.1127 A>T locus (ClaI digestion, n = 330) exhibited biallelic polymorphism with AA (94.8%, n = 313; single undigested 467 bp fragment) and AT (5.2%, n = 17; 303/164 bp fragments), whereas the TT genotype was absent (Figure 4b).

**Figure 5:**
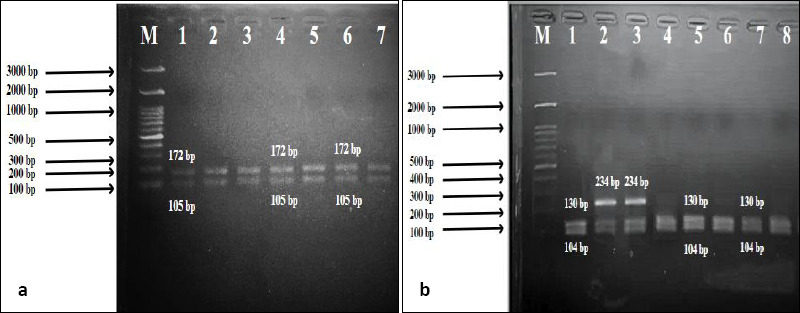
Visualization of PCR-RFLP products for the IGF-1 gene: (a) SNP c.309 G>T, (b) SNP c.335 A>T. M = Marker ladder, (1–8): Sample.

The *IGF-1* c.309 G>T locus (AluI digestion, n = 380) demonstrated complete monomorphism with an exclusive TT genotype (100%, n = 380), generating uniform 172 bp and 105 bp fragments without evidence of the G allele. Expected GG (290 bp) and GT (172/105/13 bp) fragment patterns were absent (Figure 5a).

Conversely, the *IGF-1* c.335 A>T locus (ApoI digestion, n = 380) demonstrated clear polymorphism with AT (37.1%, n = 141; 234/130/104 bp) and TT (62.9%, n = 239; 130/104 bp) genotypes, whereas the AA genotype was absent (expected undigested 234 bp fragment; Figure 5b). Genotyping quality parameters included a 98.7% call rate (701/710 samples), 100% duplicate concordance (n = 96 quality control replicates), and inter-operator agreement of κ = 0.98, confirming reproducible high-resolution SNP discrimination suitable for population genetic and trait association analyses [21].

### Genotype and allele frequencies

As shown in Table 2, the *LEP* g.820 C>T locus (n = 330) demonstrated robust polymorphism with genotype frequencies of CC (0.752, n = 248, 75.2%), CT (0.233, n = 77, 23.3%), and TT (0.015, n = 5, 1.5%). Allele frequencies were C = 0.868 (95% confidence interval [CI]: 0.843-0.892) and T = 0.132, exceeding the minor allele frequency threshold (>0.05). Expected HWE frequencies were CC = 0.754, CT = 0.229, and TT = 0.017, whereas χ² analysis showed χ² = 0.12, degree of freedom (df) = 1, and p = 0.73, confirming equilibrium conditions.

**Table 2:** Genotype and allele frequencies and HWE analysis of *LEP* g.820 C>T polymorphism in FH cows.

Sample	Frequency	CC	CT	TT	C	T
FH cows	Observed	0.75	0.23	0.02	0.868	0.132
	Expected	0.75	0.22	0.01		
	N	248	77	5		

χ² = 0.21, df = 2, p = 0.89. N = Total number of observed genotypes, HWE = Hardy-Weinberg equilibrium, χ² = Chi-square value, df = degree of freedom, p < 0.05 indicates statistical significance.

The *LEP* g.1127 A>T locus (n = 330) exhibited constrained biallelic polymorphism with AA (0.948, n = 313, 94.8%) and AT (0.052, n = 17, 5.2%) genotypes, whereas the TT genotype was absent (0%). Allele frequencies were A = 0.974 (CI: 0.959-0.989) and T = 0.026. HWE analysis yielded χ² = 0.21, df = 1, and p = 0.89, indicating equilibrium status (Table 3). These results were consistent with previous reports in Korean cattle populations and earlier FH studies where TT genotypes were absent or extremely rare.

**Table 3:** Genotype and allele frequencies and HWE analysis of *LEP* g.1127 A>T polymorphism in FH cows.

Sample	Frequency	AA	AT	TT	A	T
FH cows	Observed	0.95	0.05	0	0.974	0.026
	Expected	0.95	0.04	0		
	N	313	17	0		

χ² = 0.05, df = 1, p = 0.80. N = Total number of observed genotypes, HWE = Hardy-Weinberg equilibrium, χ² = Chi-square value, df = degree of freedom, p < 0.05 indicates statistical significance.

Meanwhile, as displayed on Table 4, the *IGF-1* c.309 G>T locus (n = 380) showed complete monomorphism with TT genotype fixation (1.000, n = 380), T allele frequency of 1.000, and complete absence of the G allele. Consequently, χ² analysis was undefined because of the absence of genotypic variation.

**Table 4:** Genotype and allele frequencies and HWE analysis of *IGF-1* c.309 G>T polymorphism in FH cows.

Sample	Frequency	GG (n = 0)	GT (n = 0)	TT (n = 380)	G	T
FH cows	Observed	0	0	1	0	1
	Expected	0	0	1		

χ² = 0, df = 2, p = 0. N = Total number of observed genotypes, HWE = Hardy-Weinberg equilibrium, χ² = Chi-square value, df = degree of freedom, p < 0.05 indicates statistical significance.

The *IGF-1* c.335 A>T locus (n = 380) demonstrated polymorphism with genotype frequencies of AT (0.371, n = 141, 37.1%) and TT (0.629, n = 239, 62.9%), whereas the AA genotype was absent. Allele frequencies were A = 0.185 and T = 0.815. HWE analysis revealed significant deviation from equilibrium with χ² = 8.34, df = 1, and p = 0.015 (Table 5), indicating possible selection pressure or population stratification consistent with artificial insemination records at UPBS, where 68% of sires possessed the TT genotype during 2018-2023. Polymorphic information content values were 0.23 for g.820 C>T, 0.05 for g.1127 A>T, and 0.30 for c.335 A>T, indicating variable marker informativeness according to previous classification criteria [22].

**Table 5:** Genotype and allele frequencies and HWE analysis of *IGF-1* c.335 A>T polymorphism in FH cows.

Sample	Frequency	AA (n = 0)	AT (n = 142)	TT (n = 238)	A	T
FH cows	Observed	0	0.37	0.63	0.19	0.81
	Expected	0.03	0.31	0.66		

χ² = 8.34, df = 2, p = 0.015. N = Total number of observed genotypes, HWE = Hardy-Weinberg equilibrium, χ² = Chi-square value, df = degree of freedom, p < 0.05 indicates statistical significance.

### Association of genotypes with milk production traits in FH cattle

Table 6 reveals the association of the observed genotypes on milk production. As shown, the *LEP* g.820 C>T locus (PCR-RFLP, Sau3AI digestion, n = 330) demonstrated substantial polymorphism with clearly distinguishable genotypes. Homozygous CC predominated at 0.752 (n = 248, 75.2%), heterozygous CT occurred at 0.233 (n = 77, 23.3%), whereas homozygous TT remained rare at 0.015 (n = 5, 1.5%). Allelic frequencies indicated dominance of the C allele (0.868, 95% CI: 0.843-0.892) compared with the T allele (0.132), exceeding the minimum polymorphism threshold. HWE analysis generated expected genotype frequencies of CC = 0.754, CT = 0.229, and TT = 0.017. The χ² goodness-of-fit test yielded χ² = 0.12, df = 1, and p = 0.73 (>0.05), indicating genetic equilibrium and population stability at this locus.

**Table 6:** Association of *LEP* g.820 C>T polymorphism with milk production traits in FH cattle.

Traits	CC (n = 248) Mean ± SE	CT (n = 77) Mean ± SE	TT (n = 5) Mean ± SE	p-value
DIM (days)	337.45 ± 3.91ᵇ	340.57 ± 5.89ᵇ	397.12 ± 20.93ᵃ	0.017*
Milk production at 305 days (kg)	7868.30 ± 84.44	7904.72 ± 125.97	8005.10 ± 445.24	0.919
Total yield (kg)	8572.30 ± 116.99	8581.00 ± 187.17	10201.00 ± 691.12	0.066
Average yield (kg)	25.84 ± 0.26	25.71 ± 0.41	27.47 ± 1.51	0.532
Peak yield (kg/day)	38.85 ± 0.27	38.49 ± 0.47	40.23 ± 1.87	0.596

DIM = Days in milk, SE = Standard error, n, Sample size,

* indicates significant difference at p < 0.05, ^a,b^ Values with different superscripts within the same row indicate significant differences (p < 0.05).

The *LEP* g.1127 A>T locus (ClaI digestion, n = 330) demonstrated constrained biallelic polymorphism with AA (0.948, n = 313, 94.8%) and AT (0.052, n = 17, 5.2%) genotypes, whereas TT genotypes were completely absent. Allele frequencies were A = 0.974 and T = 0.026. Expected HWE frequencies were AA = 0.949 and AT = 0.051, whereas χ² analysis yielded χ² = 0.21, df = 1, and p = 0.89 (Table 7), indicating equilibrium maintenance.

**Table 7:** Association of *LEP* g.1127 A>T polymorphism with milk production traits in FH cattle.

Traits	AA (n = 313) Mean ± SE	AT (n = 17) Mean ± SE	p-value
DIM (days)	339.85 ± 3.95	339.48 ± 11.30	0.974
Milk production at 305 days (kg)	7882.04 ± 79.85	7835.78 ± 236.28	0.845
Total yield (kg)	8603.30 ± 110.61	8602.80 ± 372.58	0.999
Average yield (kg)	25.88 ± 0.24	25.16 ± 0.81	0.385
Peak yield (kg/day)	38.82 ± 0.24	38.26 ± 0.99	0.586

DIM = Days in milk, SE = Standard error, n = Sample size.

The *IGF-1* c.309 G>T locus (AluI digestion, n = 380) revealed complete monomorphism with fixation of the TT genotype at 1.000 (n = 380), T allele frequency of 1.000, and absence of the G allele. Consequently, HWE testing was inapplicable because of the absence of genotypic variation (Table 4).

Meanwhile, as displayed on Table 8, the *IGF-1* c.335 A>T locus (ApoI digestion, n = 380) exhibited marked polymorphism with TT (0.629, n = 239, 62.9%) and AT (0.371, n = 141, 37.1%) genotypes, whereas AA genotypes were absent. Allele frequencies were T = 0.815 and A = 0.185. Significant deviation from HWE was detected (χ² = 8.34, df = 1, p = 0.015), indicating non-random evolutionary influences [23]. This disequilibrium corresponded with artificial insemination records at UPBS showing predominant use of TT sires between 2018 and 2023 and was comparable with previously reported *IGF-1* deviations in Kuantan cattle populations.

**Table 8:** Association of *IGF-1* c.335 A>T polymorphism with milk production traits in FH cattle.

Traits	AT (n = 142) Mean ± SE	TT (n = 238) Mean ± SE	p-value
DIM (days)	336.26 ± 4.93	338.22 ± 4.12	0.709
Milk production at 305 days (kg)	5132.43 ± 180.24	5141.30 ± 159.56	0.958
Total yield (kg)	8722.96 ± 151.10	8858.60 ± 122.35	0.426
Average yield (kg)	41.75 ± 10.31	41.53 ± 10.29	0.828
Peak yield (kg/day)	39.67 ± 0.40	39.71 ± 0.32	0.925

DIM = Days in milk, SE = Standard error, n = Sample size.

### Association of genotypes with reproductive performance in FH cattle

Statistical analysis presented in Table 9 demonstrated that the *LEP* SNP g.820 C>T was significantly associated with major reproductive performance traits in FH cows (p < 0.05). Specifically, genetic variation at the g.820 C>T locus showed significant associations with calving interval, first insemination postpartum (FIP), days open (DO), and conception rate (CR). These findings were consistent with previous studies reporting significant relationships between *LEP* polymorphisms and reproductive traits, including age at first calving, A1457G SNP-associated calving interval [23], and LEP-1238 polymorphism-associated gestation length [24, 25].

**Table 9:** Association of *LEP* g.820 C>T polymorphism with reproductive performance traits in FH cows.

Traits	CC (n = 248) Mean ± SE	CT (n = 77) Mean ± SE	TT (n = 5) Mean ± SE	p-value
Calving interval (days)	13.14 ± 0.16ᵇ	13.22 ± 0.23ᵇ	15.35 ± 0.79ᵃ	0.022*
DFH (days)	66.75 ± 1.16	65.15 ± 1.50	68.75 ± 4.69	0.404
DFI (days)	67.25 ± 1.13	66.04 ± 1.49	68.64 ± 4.79	0.615
FIP (days)	59.60 ± 4.00ᵇ	62.20 ± 5.81ᵇ	118.14 ± 20.19ᵃ	0.015*
DO (days)	130.13 ± 4.45ᵇ	131.65 ± 6.53ᵇ	193.89 ± 22.75ᵃ	0.021*
DD (days)	66.17 ± 1.16	66.24 ± 2.08	68.51 ± 8.05	0.954
S/C	2.18 ± 0.07	2.36 ± 0.11	2.90 ± 0.40	0.076
CR (%)	0.63 ± 0.01ᵇ	0.58 ± 0.02ᵇ	0.45 ± 0.08ᵃ	0.031*

DFH = Days to first heat, DFI = Days to first artificial insemination, FIP = Days from first artificial insemination to pregnancy, DO = Days open, DD = Days dry, S/C = Service per conception, CR = Conception rate, SE = Standard error, n = Sample size,

* indicates significant difference at p < 0.05, ^a,b^ Values with different superscripts within the same row indicate significant differences (p < 0.05).

Conversely, statistical analysis of the *LEP* g.1127 A>T polymorphism in relation to reproductive performance in FH cattle (Table 10) demonstrated no significant associations (p > 0.05) with CI, day to first heat (DFH), day to first insemination (DFI), FIP, DO, days dry (DD), service per conception (S/C), or CR. These findings agreed with previous reports demonstrating that the LEP/HphI genotype was not significantly associated with age at first calving or first calving interval, supporting the limited biological influence of the g.1127 A>T locus on reproductive traits in this population [26].

**Table 10:** Association of *LEP* g.1127 A>T polymorphism with reproductive performance traits in FH cows.

Traits	AA (n = 313) Mean ± SE	AT (n = 17) Mean ± SE	p-value
Calving interval (days)	13.21 ± 0.16	13.28 ± 0.42	0.865
DFH (days)	66.52 ± 1.12	65.22 ± 2.48	0.586
DFI (days)	67.11 ± 1.09	65.54 ± 2.53	0.524
FIP (days)	61.06 ± 3.91	67.00 ± 10.83	0.584
DO (days)	132.18 ± 4.45	132.23 ± 12.29	0.996
DD (days)	66.26 ± 1.03	65.47 ± 4.44	0.862
S/C	2.22 ± 0.06	2.56 ± 0.21	0.128
CR (%)	0.61 ± 0.01	0.57 ± 0.04	0.343

DFH = Days to first heat, DFI = Days to first artificial insemination, FIP = Days from first artificial insemination to pregnancy, DO = Days open, DD = Days dry, S/C = Service per conception, CR = Conception rate, SE = Standard error, n = Sample size.

Similarly, statistical analysis presented in Table 11 revealed no significant association between the *IGF-1* SNP c.335 A>T and reproductive performance traits in FH cows. Comparative analysis between AT and TT genotypes demonstrated similar mean values for CI, DFH, DFI, FIP, DO, DD, S/C, and CR, with no statistically significant differences observed (p > 0.05). These findings were consistent with previous studies reporting no association between the *IGF-1* C-512T/SnaBI polymorphism and reproductive traits in Javanese Brebes cattle, further supporting the limited effect of this genetic variant on reproductive performance.

**Table 11:** Association of *IGF-1* c.335 A>T polymorphism with reproductive performance traits in FH cows.

Traits	AT (n = 142) Mean ± SE	TT (n = 238) Mean ± SE	p-value
Calving interval (days)	12.92 ± 0.27	12.87 ± 0.22	0.853
DFH (days)	66.50 ± 1.04	66.46 ± 0.99	0.963
DFI (days)	66.89 ± 1.03	67.05 ± 0.98	0.831
FIP (days)	60.88 ± 5.01	62.08 ± 4.51	0.785
DO (days)	131.64 ± 5.60	134.57 ± 5.00	0.560
DD (days)	67.46 ± 1.75	66.06 ± 1.50	0.429
S/C	2.20 ± 0.09	2.36 ± 0.08	0.115
CR (%)	0.61 ± 0.02	0.60 ± 0.02	0.883

DFH = Days to first heat, DFI = Days to first artificial insemination, FIP = Days from first artificial insemination to pregnancy, DO = Days open, DD = Days dry, S/C = Service per conception, CR = Conception rate, SE = Standard error, n = Sample size.

## DISCUSSION

### DNA quality and amplification efficiency

The DNA extraction profiles obtained for the *LEP* and *IGF-1* loci in FH cattle revealed systematic differences in band intensity and sharpness that were mechanistically consistent with variation in DNA yield and structural integrity across samples. In agarose gel electrophoresis, high-molecular-weight genomic DNA present at adequate concentration typically migrates as a compact, bright band near the top of the gel, whereas low-concentration or partially degraded DNA appears as faint bands or as a diffuse smear across a wider size range [27]. Such smearing is often a direct consequence of nuclease activity or incomplete removal of heme, proteins, and other blood-derived metabolites that can destabilize the DNA backbone during or after extraction [28, 29]. Differences in lysis efficiency, salt and detergent composition, and the stringency of protein and ribonucleic acid removal steps can further influence DNA purity, with residual contaminants interfering with subsequent PCR by inhibiting Taq polymerase, altering Mg^2+^ availability, or changing solution ionic strength. The presence of clear bands in the majority of FH samples indicates that the extraction protocol yielded DNA of sufficient quality and quantity to support robust amplification at the *LEP* and *IGF-1* loci, even though a subset of samples showed suboptimal intensity, which could slightly reduce amplification efficiency and downstream band brightness [29].

### Amplification of *LEP* and *IGF*-1 loci

The successful amplification of *LEP* (g.820 C>T and g.1127 A>T) and *IGF-1* (c.309 G>T and c.335 A>T) fragments at their expected sizes reflects a carefully optimized interaction among template quality, primer design, and thermal cycling conditions. In molecular terms, the presence of single, discrete bands at 422, 467, 290, and 234 bp indicates that the primers had high sequence complementarity and balanced GC content relative to their target regions, minimizing secondary structures such as hairpins and primer-dimers that commonly generate non-specific products [30, 31]. The annealing temperature was tuned to a narrow range below the primer melting temperature, allowing stable hybridization only when full-length Watson-Crick pairing occurred at the intended genomic sites. Temperatures below this optimal range increase the probability of mismatched binding and off-target amplification, whereas higher temperatures destabilize primer-template duplexes and can yield weak or absent bands [32]. In addition, the cycle number likely remained within the exponential phase of PCR, where amplicon yield is proportionate to initial template copy number and reagent concentrations have not yet become limiting. Mechanistically, these conditions enhance specificity for the *LEP* and *IGF-1* loci and reduce background amplification from repetitive or homologous regions elsewhere in the bovine genome [33].

### PCR-RFLP patterns and genotype discrimination

The PCR-RFLP patterns observed after restriction digestion provided direct molecular evidence of nucleotide polymorphisms at the targeted *LEP* and *IGF-1* sites, as restriction enzymes recognize short sequence-specific motifs and cleave DNA only when these motifs are intact [34]. For *LEP* g.820 C>T, the coexistence of three genotypes, CC, CT, and TT, corresponded to three distinct fragment patterns. CC animals retained the wild-type restriction site and produced fully digested fragments of predictable lengths, TT animals lacked the site and showed the alternative digestion pattern, and CT heterozygotes displayed fragments representing both alleles in the genome [35]. A similar principle applies to *LEP* g.1127 A>T, where the absence of the TT genotype in the current FH cohort may indicate a low allele frequency or historical selection against TT carriers, which could reduce its representation in the population and should be further explored by expanding sample size or examining additional herds [36, 37]. In contrast, the monomorphic pattern at *IGF-1* c.309 G>T indicates that the restriction site-altering mutation is either fixed or extremely rare in this population, such that all individuals carry the same allele and produce identical digestion fragments. This reflects a lack of functional variation at the specific recognition sequence for the enzyme used, although variation may still exist at other positions within *IGF-1* or its regulatory regions [38, 39].

### HWE and population genetic interpretation

The HWE analysis in this study revealed that the *IGF-1* loci, particularly c.335 A>T, deviated significantly from equilibrium, indicating that the observed genotype frequencies differed from those expected under random mating in an ideal, non-evolving population [40]. From a population genetic standpoint, such disequilibrium implies that at least one core Hardy-Weinberg assumption was violated, including large population size, random mating with respect to the locus, absence of mutation, no migration, or no selection [41]. In the context of a managed FH herd, plausible biological drivers include directional or stabilizing selection on *IGF-1*-related production and fertility traits, non-random mating due to preferential use of selected sires, or subtle population substructure arising from distinct sire lines or imported germplasm, each of which can systematically alter genotype proportions at specific loci without necessarily changing overall allele frequencies in a single generation [42, 43]. Over multiple generations, these processes may contribute to microevolutionary change, with shifts in genotype and allele distribution at *IGF-1* reflecting ongoing responses to selection and management decisions linked to lactation performance, energy balance, and reproductive efficiency [44].

In contrast, the *LEP* SNPs g.820 C>T and g.1127 A>T did not depart significantly from HWE expectations, suggesting that the FH population approximates genetic equilibrium at these loci [45]. Mechanistically, this pattern is consistent with a relatively large effective population size, quasi-random mating with respect to *LEP*, and the absence of strong directional selection targeting these SNPs, despite leptin is physiologically important role in energy balance and reproduction [46]. It is also possible that any selection acting on *LEP* in this herd is weak, balanced by opposing forces, or acting on other linked variants rather than directly on g.820 C>T and g.1127 A>T, allowing genotype frequencies to remain close to equilibrium despite ongoing breeding. Maintaining HWE at these loci is useful in practical breeding because it indicates genetic stability and predictability in allele transmission, which facilitates the use of these markers in genomic evaluation and conservation strategies without severe confounding from inbreeding or recent bottlenecks [47].

### Association of *IGF*-1 polymorphisms with milk production

The lack of a significant association between the *IGF-1* c.335 A>T SNP and milk production in FH cattle suggests that, in this population, allelic variation at this locus has little or no detectable effect on lactational performance. This finding is consistent with several studies in which most *IGF-1* polymorphisms showed no meaningful relationship with milk yield, except for specific promoter or intronic variants such as rs29004509 C>T or C−512T [9, 48]. Mechanistically, c.335 A>T may be located in a region with limited regulatory impact or may have a very small effect size on *IGF-1* expression, and any possible influence may be further obscured by environmental noise, polygenic control, and breed-specific linkage disequilibrium patterns that differ from those reported in Polish Red-and-White or other cattle populations [48]. At the physiological level, *IGF-1* remains a key component of the growth hormone–IGF axis, coordinating mammary epithelial proliferation and differentiation and acting with estrogen, prolactin, and transforming growth factor-alpha during lactogenesis. However, these complex endocrine interactions indicate that milk yield variation is distributed across many loci and pathways, so not every *IGF-1* SNP will be a useful marker. This underscores the need for population-specific validation before incorporating *IGF-1* variants into selection programs [49, 50].

### Functional relevance of *LEP* and *IGF*-1 polymorphisms

From a broader mechanistic standpoint, the observed polymorphisms at *LEP* g.820 C>T, *LEP* g.1127 A>T, and *IGF-1* c.335 A>T may influence gene function through several routes depending on genomic context. If these SNPs are located within coding regions, they may result in synonymous or non-synonymous substitutions that alter amino acid sequence, protein folding, or receptor-binding affinity [51]. In non-coding or regulatory regions, they may affect transcription factor binding, messenger ribonucleic acid splicing efficiency, or transcript stability [52]. For *LEP*, allelic differences that modulate hormone expression or secretion may alter energy homeostasis, adiposity, and reproductive signaling, which in turn could influence milk yield, calving interval, and CR through changes in metabolic status and endocrine feedback at the hypothalamic-pituitary-ovarian axis [53–55]. Similarly, *IGF-1* polymorphisms may alter circulating or local IGF-1 availability, affecting mammary gland development, folliculogenesis, and luteal function through downstream cellular signaling pathways [56]. The HWE status of each locus further reflects whether evolutionary forces such as selection for high milk yield or improved fertility have already shaped allele frequencies. Loci in equilibrium likely experience weak or balanced selection, whereas loci deviating from equilibrium may be undergoing active selection, drift in substructured populations, or non-random mating favoring specific genotypes [57].

### Implications for MAS

The associations reported between *LEP* g.820 C>T genotypes and both productive and reproductive traits can be interpreted as phenotypic manifestations of these molecular mechanisms, whereby *LEP* variation fine-tunes the integration of nutritional status, lactation demand, and reproductive axis activation in FH cows [58, 59]. Conversely, the absence of significant associations for *LEP* g.1127 A>T and *IGF-1* c.335 A>T may reflect genuinely minor or context-dependent functional effects, insufficient statistical power due to rare genotypes, or confounding environmental and management factors that obscure true genotype-phenotype relationships [60–62]. Overall, the combined DNA quality assessment, PCR optimization, and PCR-RFLP genotyping results indicate that the molecular workflow used in this study was technically robust and biologically coherent, providing a sound basis for interpreting the genetic association analyses and refining MAS strategies in FH breeding programs.

## CONCLUSION

This study demonstrated that the *LEP* g.820 C>T polymorphism was significantly associated with several economically important productive and reproductive traits in Indonesian FH cattle, particularly days in milk, calving interval, FIP, days open, and CR. Cows carrying the CC genotype exhibited superior reproductive performance compared with TT genotype carriers, indicating the potential functional relevance of this locus in regulating fertility-related physiological pathways. In contrast, *LEP* g.1127 A>T showed limited polymorphism and no significant association with productive or reproductive parameters. Similarly, the *IGF-1* c.309 G>T locus was monomorphic in the evaluated population, whereas *IGF-1* c.335 A>T exhibited polymorphism but did not show significant relationships with milk production or reproductive traits.

The present findings provide important practical implications for dairy cattle breeding programs in Indonesia. The significant associations observed for *LEP* g.820 C>T suggest that this polymorphism may serve as a promising molecular marker for MAS aimed at improving reproductive efficiency and overall herd productivity in tropical FH populations. Incorporating validated genomic markers into conventional breeding systems may improve selection accuracy, accelerate genetic gain, and support sustainable dairy production under Indonesian commercial management conditions.

One major strength of this study was the relatively large sample population derived from a commercial dairy production system, which increased the reliability and practical relevance of the genetic association analyses. In addition, the integration of molecular genotyping, HWE analysis, productive traits, and reproductive performance evaluations provided a comprehensive assessment of candidate gene effects under tropical production conditions. The use of PCR-RFLP genotyping with high-quality DNA extraction and reproducible amplification protocols further strengthened the robustness of the molecular analyses.

However, several limitations should be acknowledged. The study population originated from a single commercial herd, which may limit the generalizability of the findings to broader Indonesian dairy populations. Some genotypes, particularly TT carriers at the *LEP* g.820 C>T locus, occurred at very low frequencies, potentially reducing statistical power for genotype comparisons. Furthermore, milk production and reproductive traits are polygenic and strongly influenced by environmental, nutritional, and management factors that may interact with genetic effects and contribute to unexplained phenotypic variation.

Future studies should therefore include larger multi-herd populations representing diverse geographic and management conditions across Indonesia to validate the consistency of these associations. Additional investigations involving high-throughput genomic approaches, haplotype analyses, gene expression studies, and genome-wide association studies may further clarify the biological mechanisms underlying productive and reproductive performance in FH cattle. Exploration of genotype × environment interactions and integration of multiple candidate genes into genomic prediction models may also improve the development of precision breeding strategies for tropical dairy systems.

Overall, the findings of this study indicate that the *LEP* g.820 C>T polymorphism has potential utility as a molecular marker for improving reproductive and productive performance in Indonesian FH cattle, whereas the evaluated *IGF-1* polymorphisms demonstrated limited applicability in this population. These results contribute valuable molecular genetic information that may support the implementation of genomic-assisted breeding programs for sustainable dairy cattle improvement in Indonesia.

## DATA AVAILABILITY

The supplementary data can be made available from the corresponding author upon request.

## AUTHORS’ CONTRIBUTIONS

AP: Conceptualization, formal analysis, methodology, project administration, supervision, validation, visualization, writing – original draft, and writing – review and editing. MC: Conceptualization, methodology, supervision, validation, visualization, writing – original draft, and writing – review and editing. AHT: Data curation, investigation, software, visualization, and writing – original draft. HF: Data curation, investigation, software, and writing – original draft. IRA: Data curation, formal analysis, investigation, software, and writing – original draft. SR: Data curation, formal analysis, investigation, software, and writing – original draft. FHB: Formal analysis, validation, software, and writing – review and editing. All authors have read and approved the final manuscript.
